# Preparation of cationized gelatin nanospheres incorporating molecular beacon to visualize cell apoptosis

**DOI:** 10.1038/s41598-018-33231-2

**Published:** 2018-10-04

**Authors:** Yuki Murata, Jun-ichiro Jo, Yasuhiko Tabata

**Affiliations:** Laboratory of Biomaterials, Institute for Frontier Life and Medical Sciences, Kyoto University, 53 Kawara-cho Shogoin, Sakyo-ku, Kyoto 606-8507 Japan

## Abstract

The objective of this study is to prepare cationized gelatin nanospheres (cGNS) incorporating a molecular beacon (MB), and visualize cellular apoptosis. Two types of MB to detect the messenger RNA (mRNA) of glyceraldehyde-3-phosphate dehydrogenase (GAPDH) (GAP MB), and caspase-3 (casp3 MB) were incorporated in cGNS, respectively. MB incorporated in cGNS showed the DNA sequence specificity in hybridization. The cGNS incorporation enabled MB to enhance the stability against nuclease to a significantly great extent compared with free MB. The cGNS incorporating GAP MB were internalized into the KUM6 of a mouse bone marrow-derived stem cell by an endocytotic pathway. The cGNS were not distributed at the lysosomes. After the incubation with cGNS, the cell apoptosis was induced at different concentrations of camptothecin. No change in the intracellular fluorescence was observed for cGNS_GAPMB_. On the other hand, for the cGNS_casp3MB_, the fluorescent intensity significantly enhanced by the apoptosis induction of cells. It is concluded that cGNS incorporating MB is a promising system for the visualization of cellular apoptosis.

## Introduction

Cell transplantation is one of the promising therapies in regenerative medicine to induce the regeneration and repairing of damaged tissues and organs^1–3^. For the development of cell transplantation, the non-invasive technologies and methodologies to visualize the localization, distribution, and biological functions of cells transplanted in the living body are highly required. Imaging technologies are effective in non-invasively evaluating the localization and distribution of cells transplanted^4–7^. Various therapeutic effects have been reported based on the biological functions of cells transplanted, although the cell functions are not always clear^8–10^. On the other hand, it is reported that the majority of cells transplanted undergo apoptosis^11^. Under these circumstances, it is of prime importance to non-invasively visualize the apoptosis of cells transplanted which is a key to evaluate the therapeutic efficacy.

Cellular biological functions are regulated by the concentrations and time profiles of intracellular enzymes, its coding genes, and messenger RNA (mRNA). For the detection of cellular biological functions, activatable imaging probes will be used to visualize the biological function depending on the change of intracellular environment without the cell destruction^12–14^. Molecular beacon (MB) is a mRNA detectable activatable probe of a stem-loop structured nucleic acid derivative, and consisted of 25 to 30 bases^15,16^. The quencher and fluorophore are conjugated at both the end sides of MB. In the absence of the target mRNA, MB is in the quenched state. In contrast, in the presence of the target mRNA, the structure of MB is changed to be fluorescent based on the hybridization with the target mRNA. Based on the system, MB can detect the target mRNA in response to the intracellular concentration of target mRNA^17–19^.

Gelatin is a biodegradable polymer, and the bio-safety and biocompatibility have been proved by the long-term food, medical, and pharmaceutical applications. Various shapes of gelatin hydrogels can achieve the controlled release of proteins and low-molecular weight drugs^20–22^. Cationized gelatin can readily be prepared by simply introducing amine residues to the carboxyl groups of gelatin. Cationized gelatin hydrogels enabled the controlled release of plasmid DNA (pDNA) and small interfering RNA (siRNA)^23,24^. In addition, cationized gelatin nanospheres achieved the intracellular controlled release of pDNA^25^, and siRNA^26^. On the other hand, gelatin nanospheres are also applicable to the carrier of an imaging probe^27^. In this study, the cationized gelatin nanospheres are used as the carrier of MB to allow the internalization into cells.

The objective of this study is the preparation of cationized gelatin nanospheres (cGNS) incorporating MB aiming at the visualization of cell apoptosis. Two types of MB are used. One is glyceraldehyde-3-phosphate dehydrogenase (GAPDH) MB which can detect GAPDH mRNA of a housekeeping gene constantly expressing in the cells. The other is caspase-3 MB which can detect caspase-3 mRNA of an apoptosis target expressing in apoptotic cells. The cGNS preparation was performed in different conditions to optimize their physicochemical properties for cellular internalization. The mRNA sequence specificity in hybridization and the stability of cGNS_MB_ against nuclease were evaluated. The cytotoxicity, cellular internalization, and intracellular localization of cGNS_MB_ were investigated. The fluorescent intensity change of cells incubated with the cGNS_casp3MB_ was evaluated to analyze the functional response of MB. Apoptosis was induced by the treatment of camptothecin of a common apoptosis inducer for cells which had been incubated with the cGNS_casp3MB_. We examine the cell apoptosis by the conventional gene expression of caspase-3.

## Results

### Characterization of cGNS with or without MB incorporation

**Figure 1:**
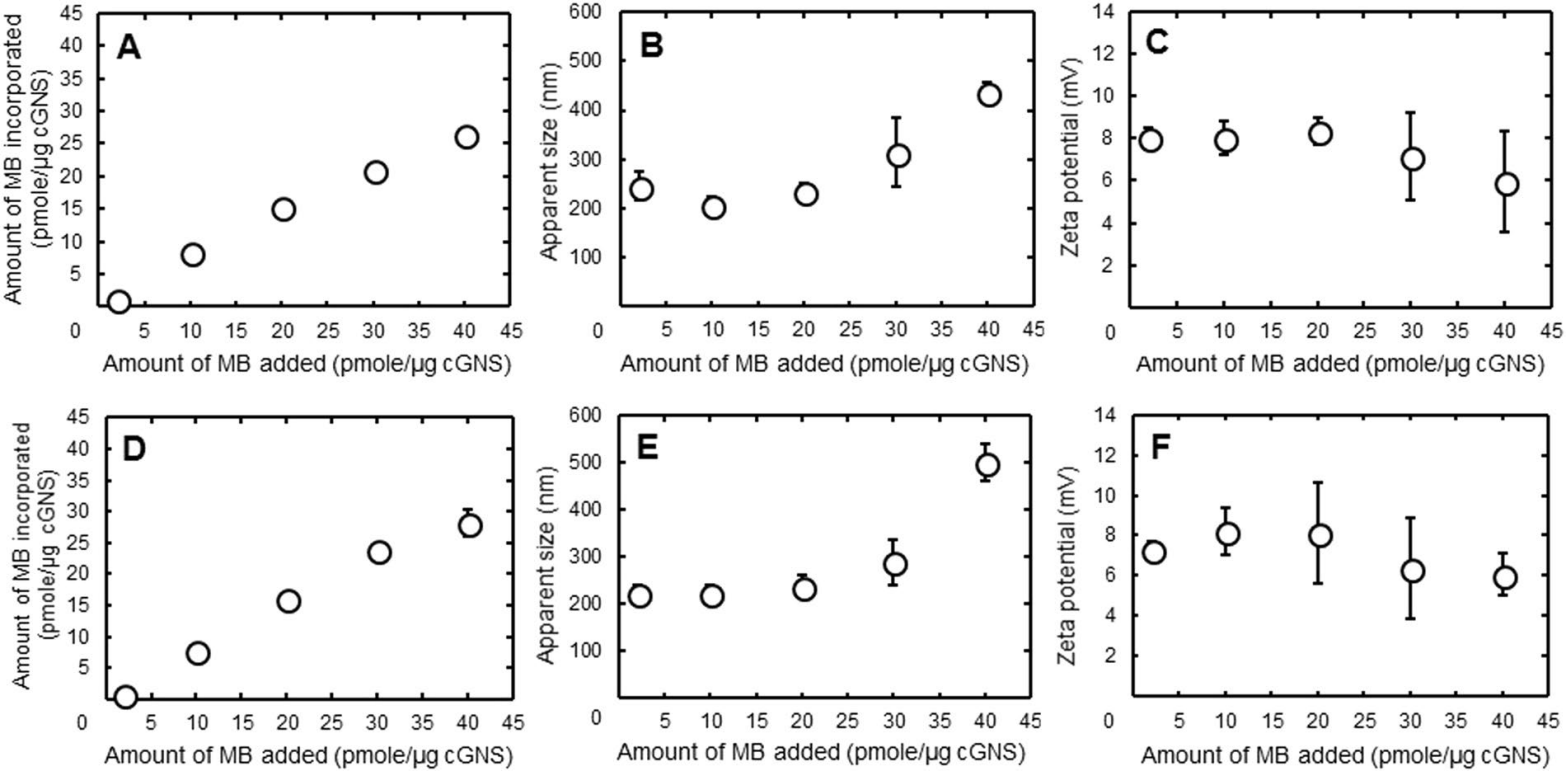
The amount of MB incorporated (**A** and **D**), apparent size (**B** and **E**), and zeta potential (**C** and **F**) of cGNS_GAPMB_ (**A**–**C**) and cGNS_casp3MB_ (**D**–**F**) prepared at different ratios of MB to cGNS.

The apparent size and zeta potential of MB-free, empty cGNS were 155.1 ± 2.8 nm and +8.18 ± 0.06 mV, respectively. Figure 1 shows the amount of MB incorporated in cGNS, the apparent size, and the zeta potential of cGNS_GAPMB_ and cGNS_casp3MB_. Both the cGNS_GAPMB_ and cGNS_casp3MB_ showed similar physicochemical properties. The amount of MB incorporated in cGNS increased as an increase of MB amount added. The apparent size also increased, whereas the zeta potential tended to decrease as the amount of MB added increased. The following experiments were performed using the nanospheres prepared at the MB amount of 20 pmole/µg cGNS.

### DNA sequence specificity and nuclease stability of MB incorporated in cGNS

**Figure 2:**
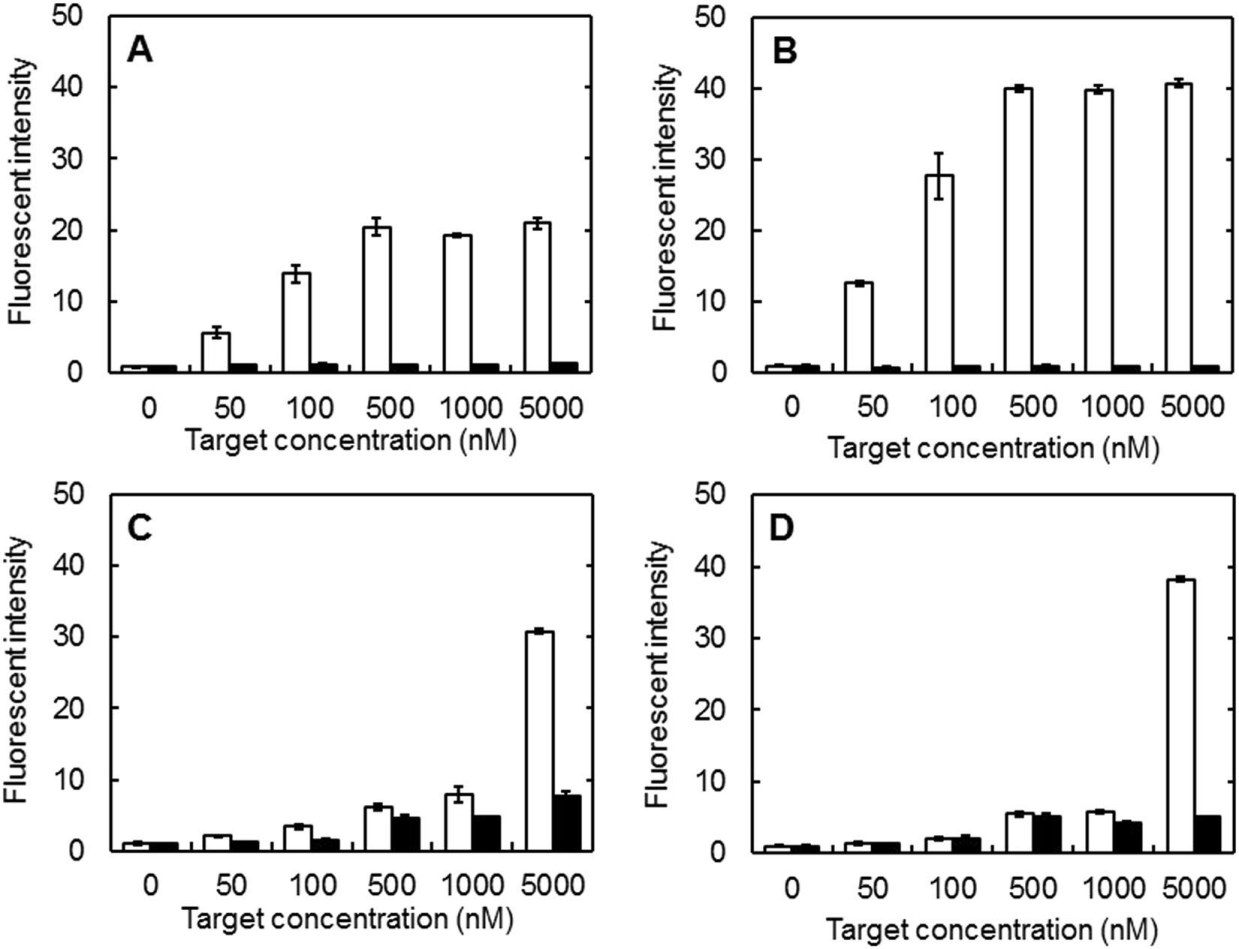
Hybridization specificity of free MB and MB incorporated in cGNS. The fluorescent intensity of free GAP MB (**A**), free casp3 MB (**B**), cGNS_GAPMB_ (**C**), and cGNS_casp3MB_ (**D**) mixed with different concentrations of specific (□) and non-specific target oligonucleotides (■). The concentration of MB is all 100 nM.

Figure 2 shows the hybridization specificity of free MB and MB incorporated in cGNS. Both the fluorescent intensities of free GAP MB and casp3 MB increased with the increased specific target concentration. On the other hand, an increase of non-specific target concentration did not affect the free MB fluorescent intensity (Fig. 2A,B). The fluorescent intensity of both GAP MB and casp3 MB incorporated in cGNS increased as the increase of specific target concentration. However, compared with free MB, the increase was small at the low concentrations of specific target. In addition, the fluorescent intensity of MB incorporated in cGNS was slightly increased as the increase of non-specific target concentration (Fig. 2C,D).

**Figure 3:**
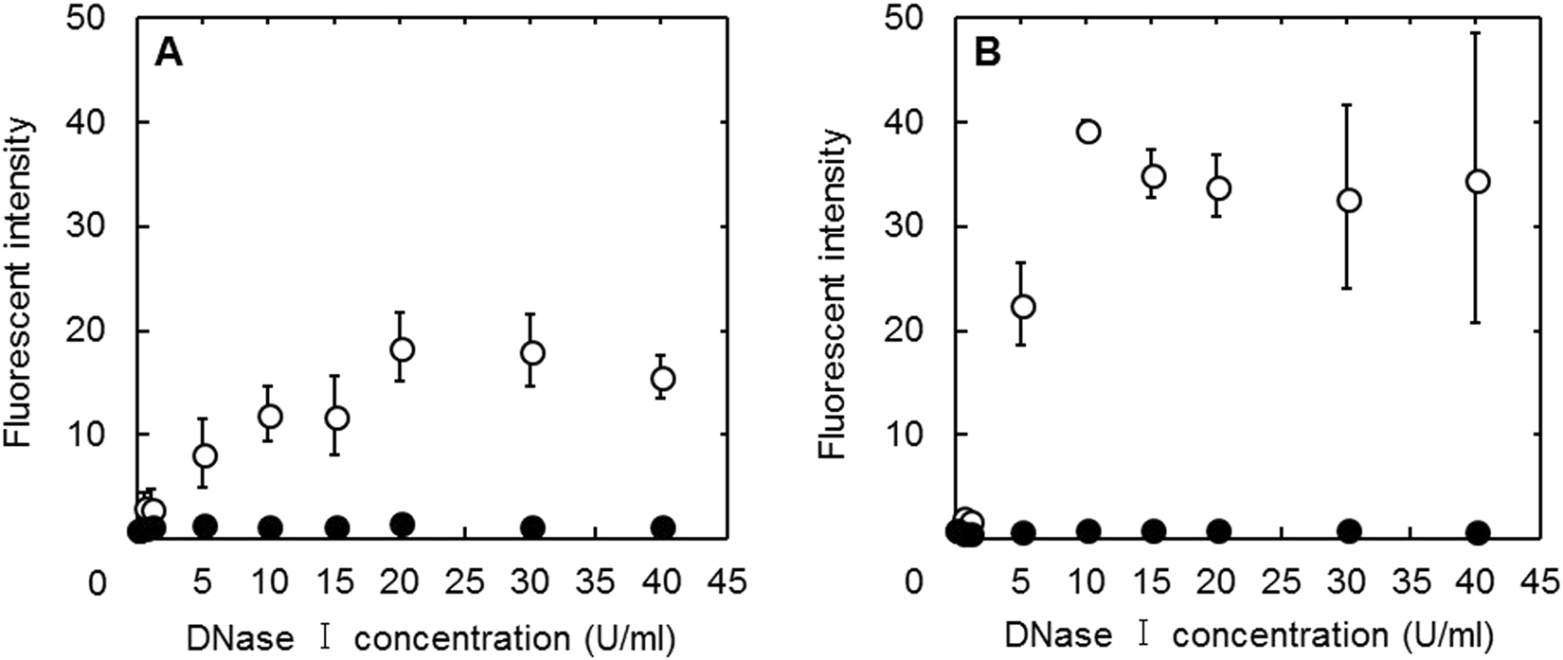
Nuclease stability of free MB (○) and MB incorporated in cGNS (●). The fluorescent intensity after mixing GAP MB (**A**) and casp3 MB (**B**) with different concentrations of DNase I. The concentration of MB is all 100 nM.

Figure 3 shows the nuclease stability of free MB and MB incorporated in cGNS. Both the free GAP MB and casp3 MB were degraded and the fluorescent intensity was increased with the DNase I concentration. On the other hand, the fluorescent intensity of both GAP MB and casp3 MB incorporated in cGNS was constant at any DNase I concentration.

### Cell viability after incubation with cGNS incorporating MB

Supplementary Fig. 1 shows the cell viability after incubation with cGNS incorporating MB. The percentage of cells survived tended to decrease as the increase of cGNS concentration for both the cGNS_GAPMB_ and cGNS_casp3MB_. Significant cytotoxicity was observed at the concentrations of 15 and 20 µg/ml.

### Cellular internalization and intracellular localization

Supplementary Fig. 2A shows the cellular internalization amount of MB incorporated in cGNS. The amount of MB internalized became higher with the increase of cGNS concentration, and reached a plateau level.

When the cellular internalization of MB incorporated in cGNS was investigated at 37 and 4 °C incubation, the amount of MB internalized at 4 °C incubation was significantly lower than that at 37 °C (Supplementary Fig. 2B). In addition, free MB were hardly internalized into the cells both at 37 and 4 °C (Supplementary Fig. 2C).

Supplementary Fig. 3 shows the cellular internalization of FITC-cGNS_GAPMB_ and the intracellular localization of cGNS or MB, lysosomes, and nuclei. Both the cGNS and MB were localized in the cells in a similar distribution pattern. Supplementary Fig. 3B(a) shows the co-localization for all the MB and cGNS. It is apparent from Supplementary Fig. 3B(b) that the MB-cGNS complexes were not distributed at the lysosomes (magenta) of cells.

### Fluorescent imaging of apoptosis

**Figure 4:**
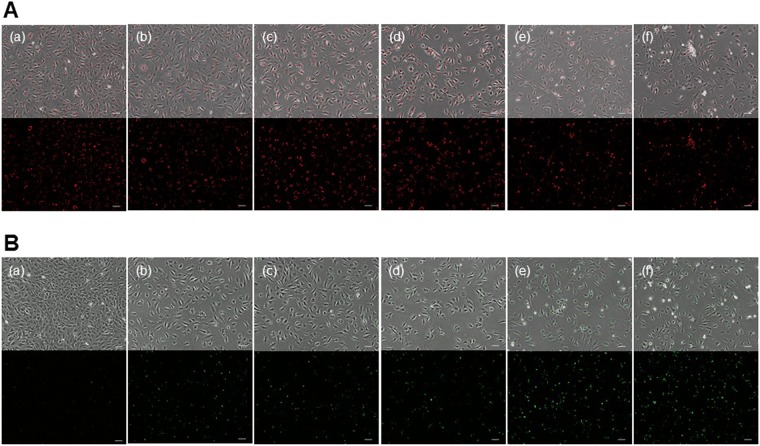
Fluorescent microscopic images of cellular apoptosis. Fluorescent images of cells incubated with 10 µg/ml cGNS_GAPMB_ (**A**) and cGNS_casp3MB_ (**B**). Fluorescent images of cells after incubation with 0 (a), 1 (b), 2 (c), 5 (d), 10 (e), and 20 µM of camptothecin (f). Scale bar is 100 µm.

**Figure 5:**
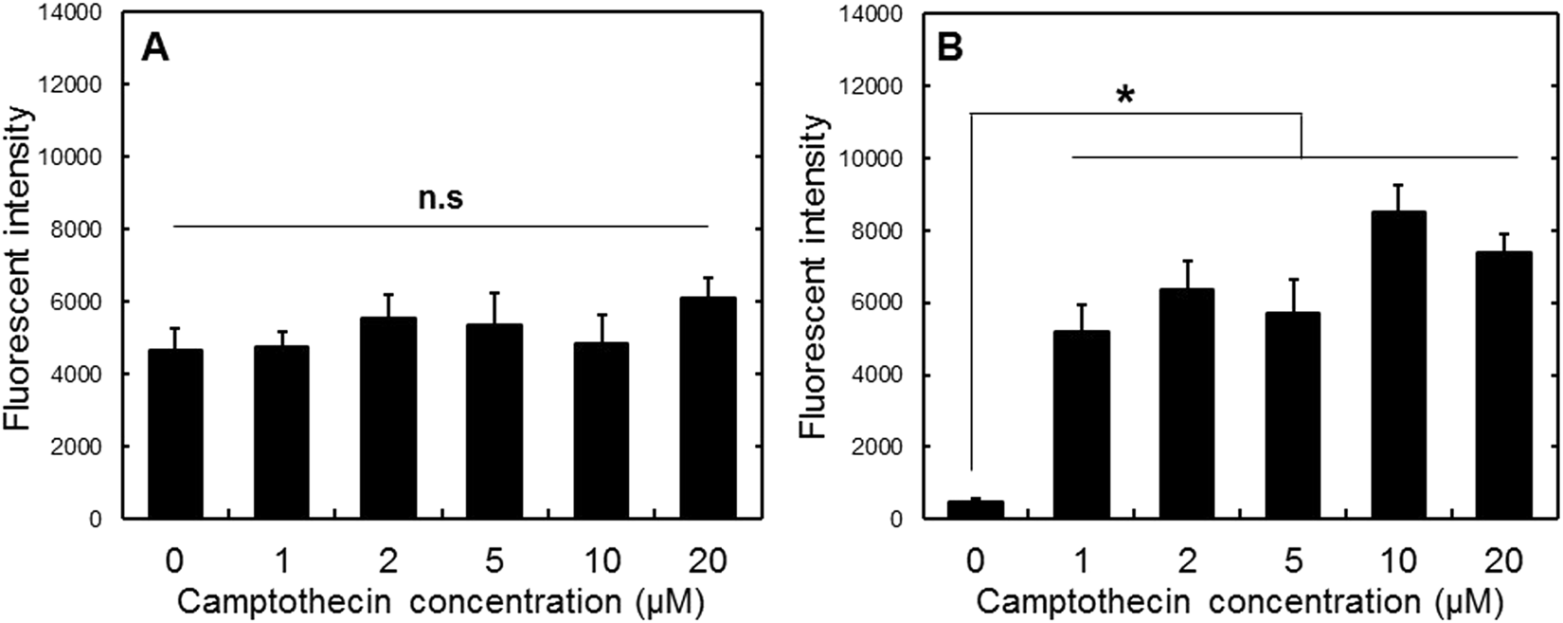
Apoptosis evaluation of cells incubated with cGNS_GAPMB_ and cGNS_casp3MB_. Fluorescent images of cells after incubation with 10 µg/ml cGNS_GAPMB_ (**A**) and cGNS_casp3MB_ (**B**). n.s; not significant. *p < 0.05; significant against the fluorescent intensity of cells at 0 µM of camptothecin.

After the cell incubation and internalization of cGNS, the cells following the addition of various concentrations of camptothecin, were observed by the fluorescent microscopy (Fig. 4). Figure 4A,B show the fluorescent images of cells incubated with cGNS_GAPMB_ and cGNS_casp3MB_, respectively. The fluorescence of GAP MB was constantly observed, irrespective of the camptothecin addition. On the contrary, for the casp3 MB, fluorescence was observed only after the camptothecin addition. Figure 5 shows the fluorescent intensity of cells incubated with cGNS_GAPMB_ and cGNS_casp3MB_ after the addition of camptothecin. The fluorescence of cells incubated with cGNS_GAPMB_ was constant at any camptothecin concentration. On the other hand, the fluorescence of cells incubated with cGNS_casp3MB_ was significantly increased after the addition of camptothecin.

### Apoptosis analysis

**Figure 6:**
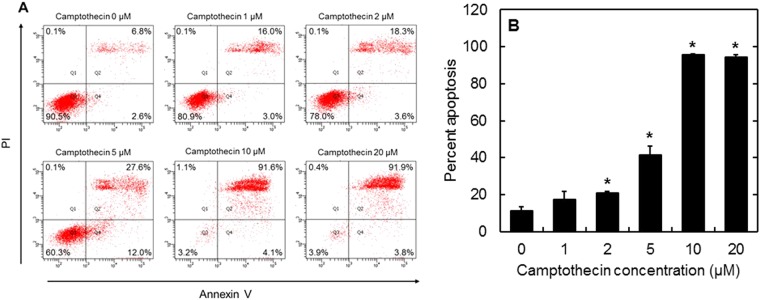
Apoptosis analysis of cells after incubation with different concentrations of camptothecin. (**A**) Flowcytometric plots of cell population and (**B**) the percentage of apoptotic cells 12 hr after incubation with different camptothecin concentrations. *p < 0.05; significant against the percent apoptosis at 0 µM camptothecin.

Figure 6 shows the flow cytometry results of the conventional apoptosis analysis. As the concentration of camptothecin increased, the number of PI and annexin V double positive cells increased, whereas PI and annexin V double negative cells decreased. At the concentrations of 10 and 20 µM, more than 90% of cells were in an apoptosis condition.

### mRNA expression analysis

**Figure 7:**
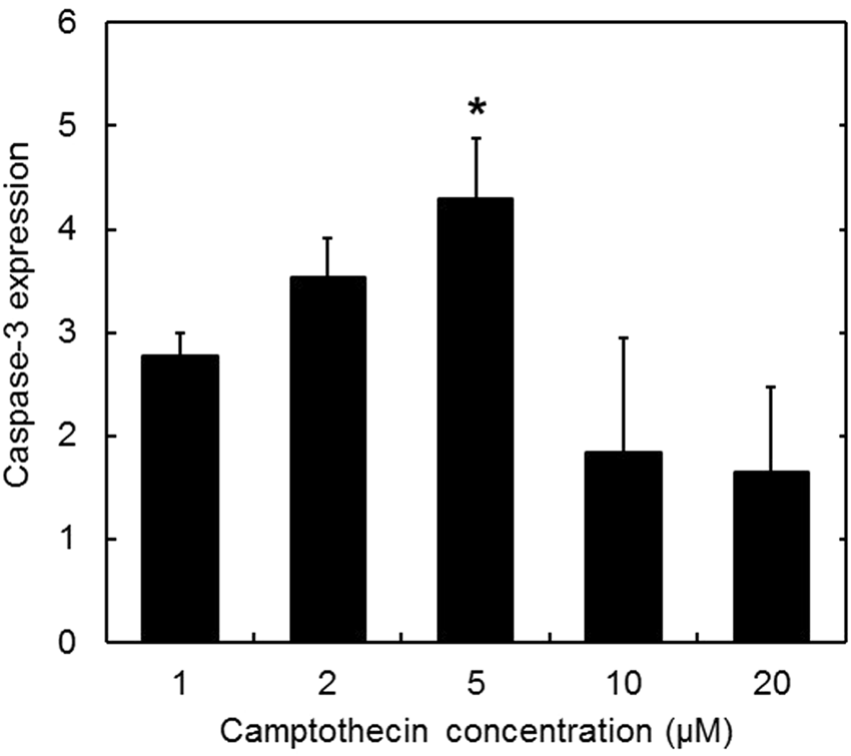
Expression analysis of caspase-3 mRNA 12 hr after incubation with different camptothecin concentrations. *p < 0.05; significant against the expression level at 0 µM camptothecin.

Figure 7 shows the expression level of caspase-3 mRNA. Compared with the original cells, the expression level increased with the increase of camptothecin concentrations. Moreover, at the concentrations of 10 and 20 µM, the expression of caspase-3 mRNA was the same level as that of the original cells at 0 µM of camptothecin.

## Discussion

The present study demonstrates that cGNS incorporating MB prepared were readily internalized into the cells as expected. The hybridization assay revealed that MB incorporated in cGNS showed the DNA sequence specificity. In addition, the higher stability against the nuclease was observed than free MB. When the apoptotic cells were prepared by the camptothecin addition, the cGNS_casp3MB_ showed the intracellular fluorescence expression according to the induction of cell apoptosis.

The mixing ratio of cGNS to MB in the cGNS preparation had an influence on the apparent size and zeta potential of cGNS (Fig. 1). An increased amount of MB added increased the apparent size, but decreased the zeta potential. The MB-free, empty cGNS have a positive zeta potential, whereas the MB have a negative charge. Therefore, it is likely that the increased amount of MB added might cause the aggregation of nanospheres due to the electrostatic interaction. The apparent size of cGNS_MB_ is larger than that of MB-free, empty cGNS. The aggregation of nanospheres might be due to the association among the nanospheres with MB. This formation change would become the increased size and decreased zeta potential. It is considered that some MB are on the surface of nanospheres, whereas the majority of MB are incorporated due to the small size of MB (31 and 32 bases of GAP MB and casp3 MB, respectively) and the constant zeta potential (+8 mV of the MB-free, empty cGNS and cGNS_MB_ prepared at 2, 10, and 20 pmole/µg cGNS). In this study, since the cGNS incorporating MB have a positive charge and a small size of 200 nm for better cellular internalization^28,29^, the cGNS prepared at the ratio of 20 pmole/µg cGNS were selected, although the MB amount increased with an increase in the MB/cGNS ratio. Similar physicochemical properties had for both the cGNS_GAPMB_ and cGNS_casp3MB_. This finding demonstrates that the physicochemical properties of cGNS incorporating MB are not influenced by the sequence of MB. Considering the biological function of MB, it is important to evaluate the sequence specificity of MB and MB incorporated in cGNS for the hybridization^30,31^. The hybridization study indicated that the fluorescent recovery of both MB and MB incorporated in cGNS was specific for the target sequences (Fig. 2). Compared with free MB, the fluorescent recovery of MB incorporated in cGNS was small at the low concentrations of specific target. This is because the incorporation of MB in the cGNS prevents the target oligonucleotides from the specific association. However, sufficient fluorescent recovery was observed at the high concentrations of specific target. It is conceivable that the higher concentration contributes to an increase in the molecular association. The reason of slight fluorescent increase of MB incorporated in cGNS with the non-specific target is unclear at present. The cationized gelatin and MB are electrostatically interacted to each other, and the negative charge of MB may be shielded by the positive charge of cationized gelatin. It is possible that this contributes to the slight increase of fluorescent intensity with non-specific target. However, at the target concentration of 5000 nM, the fluorescent intensity with the specific target was sufficiently higher than with non-specific target (4 and 7 times for GAP and casp3 MB, respectively). In addition to the hybridization specificity, the stability against nuclease is also important in terms of the cellular signal accuracy of MB^32,33^. The free MB was readily degraded depending on the DNase I concentrations. In contrast, MB incorporated in cGNS was hardly degraded by DNase I (Fig. 3). We can say with certainly that the poor approach of DNase I to MB incorporated in the cGNS, resulting in an inhibited enzymatic reaction to MB.

It is apparent in Supplementary Fig. 1 that the high concentrations of cGNS showed significant cytotoxicity. It is well known that cationic substances have a cytotoxic nature^34,35^. The amount of MB internalized into the cells increased as the increase of cGNS concentration, but reached a plateau level (Supplementary Fig. 2A). The cytotoxicity might be due to the increased cellular internalization of cGNS. However, the amount of cGNS internalized into the cells saturated over 15 µg/ml of cGNS concentration. It is possible, therefore, that an excess amount of cGNS which does not contribute to the cellular internalization, might cause the cytotoxicity. Considering the non-cytotoxic concentration, 10 µg/ml was selected for the amount of cGNS used in the experiment. In addition, 10 µg/ml of cGNS_MB_ did not affect the cell proliferation (data not shown).

The amount of MB internalized into the cells at 4 °C was significantly lower than that at 37 °C (Supplementary Fig. 2B). This suggests that cGNS incorporating MB was internalized into the cells mainly via endocytosis. On the contrary, free MB were hardly internalized both at 37 and 4 °C (Supplementary Fig. 2C), and no fluorescence was observed in the cells. This higher internalization of MB for the cGNS_MB_, so-called the concentration effect of MB, is one of the reasons to enhance the MB response in the cells. The red color of MB and the green color of cGNS were well co-localized, which can be confirmed by the yellow color in the cells (Supplementary Fig. 3B(a)). This result indicates that both the MB and cGNS were internalized in the cells in the complex state. Moreover, MB-cGNS complexes (yellow) were not co-localized with lysosomes (magenta) in the cells (Supplementary Fig. 3B(b)). This finding demonstrates that the cGNS_MB_ were endosomally escaped following the endocytosis. This may be due to the pH buffering effect by the secondary amino groups of spermine^36^. Taken together, MB-cGNS complexes internalized into the cells via endocytosis, were released into cytosol, and specifically responded with the intracellular mRNA without the degradation by the nucleases (Figs 2 and 3, Supplementary Figs 2B and 3).

In this study, camptothecin was used as an apoptosis inducer commonly used. As the mechanism to induce the cell apoptosis, it is reported that camptothecin conjugates with an DNA-topoisomerase I complex to inhibit the topoisomerase I activity^37,38^. For the cells incubated with cGNS_GAPMB_, constant fluorescence was observed, irrespective of the apoptosis induction (Figs 4 and 5). On the contrary, cGNS_casp3MB_ were not fluorescent for the original non-apoptotic cells, but after the apoptosis induction, the fluorescence was detected. This experimentally confirms that cGNS incorporating MB successfully functioned to detect the intracellular target mRNA. The fluorescent intensity of cGNS_casp3MB_ was almost constant at any concentrations of camptothecin except for 0 µM. This might be considered that the fluorescent recovery due to the hybridization of casp3 MB with the target mRNA is saturated even at low concentrations of camptothecin. For the conventional apoptosis detection, the apoptosis analysis of annexin V/PI staining was performed. PI stains the late apoptotic and dead cells because PI is only permeable to the membrane of damaged cells. The annexin V stains only the apoptotic cells because it conjugates with the phosphatidylserine of an apoptotic marker^39^. The flowcytometric assay revealed that the cell population was moved to the apoptotic fraction depending on the concentrations of camptothecin, and at the high concentrations (10 and 20 µM) more than 90% cells showed to be apoptotic (Fig. 6). The advantages of cGNS incorporating MB over the conventional apoptosis detection are to visualize the intracellular localization of apoptosis target and detect which cells are in an apoptotic condition. In addition, cGNS incorporating MB could detect the apoptotic cells after the addition of 1 and 2 µM camptothecin which were less than 20% apoptotic cell. This is also the advantage of cGNS incorporating MB due to the detection of apoptosis target mRNA which expresses in the earlier apoptotic stage. The expression level of caspase-3 mRNA evaluated by the RT-PCR (Fig. 7) tended to increase with the camptothecin concentration. However, at the concentrations of 10 and 20 µM, the expression levels were similar to those of the original cells. This is because the majority of cells after the addition of 10 and 20 µM camptothecin was in the late apoptosis state (Fig. 5), and the intracellular RNA might be degraded. On the other hand, for cGNS_casp3MB_, the increased fluorescence could be detected even after the addition of 10 and 20 µM camptothecin. The fluorescent intensity of MB was constant at the high target concentrations (Fig. 2). This finding indicates that few amount loss of intracellular target mRNA might not lead to decrease in the MB fluorescence. The conventional mRNA expression analysis by RT-PCR is not generally detect the casp3 mRNA in late apoptotic cells. On the contrary, cGNS_casp3MB_ system specifically detects the caspase-3 mRNA. This is another advantage of cGNS incorporating MB.

Recently, MB have been reported as an effective tool to visualize the cell biological functions^40–43^. The advantage of MB is of high versatility because the target is mRNA. Only by simply designing and changing the MB sequences, multiple intracellular mRNA can be detected. This detection method will be able to be universal. Various methods for the cellular internalization of MB have been reported. In general, the gene transfection techniques, such as electroporation, microinjection, sonoporation, gene gun, reversible permeabilization methods, cell-penetrating peptides, and transfection reagents, can be applied to the MB delivery into the cells^18^. These transfection techniques may be suitable to analyze the intracellular event immediately after the transfection. However, to our knowledge, most of these studies focused on the intracellular events within 24 hr after the transfection^44–46^. The long-term visualization of cellular biological functions by MB is a challenging problem. It has been reported that the intracellular MB signal was diminished by fourth day after the transfection using xtremeGENE HP™ reagent, and the continuous MB transfection was needed to monitor the osteogenesis of adipose-derived stem cells for 10 days^42^. This may be due to the easy degradation of innate MB. One strategy to prolong the MB activity is the modification of MB with locked nucleic acids (LNA)^47^ and 2′-O-methylation^33,48^. These modifications of MB based on nucleic acids chemistry allow MB to get a stability against nucleases and a higher specificity. It has been reported that 2′-O-methyl modified MB visualizing Oct-4 mRNA of a pluripotent marker were persistent in human embryonic stem cells at least for 7 days after the electroporation-based transfection^48^. In addition to the MB modifications, we think that another strategy to prolong the activity of MB is an intracellular controlled release of MB based on the material science. Cationized gelatin nanospheres (cGNS) have been reported as a carrier of pDNA and siRNA, and can achieve the sustained release of nucleic acid molecules, leading to a prolonged the bioactivity. This is based on the intracellular degradation of gelatin^25,26^. The advantage of cGNS is to allow the intracellular controlled release of MB. On the other hand, other nanocarrier-based MB delivery systems, such as poly (lactic-co-glycolic acid) (PLGA) nanoparticles, are reported^43^. We think that the advantage of cGNS over PLGA nanoparticles is the simplicity of controlling degradability and the consequent controllability of nucleic acids release profile. The controlled release of nucleic acids regulated by the gelatin degradation have been applied extracellularly^23^ and intracellularly^25,26^. Based on these reports, we strongly believe that cGNS enable the sustained release of MB, leading to the prolonged and controlled visualization of cellular biological functions. The MB release can be controlled only by the degradation of cGNS. The release profile is readily regulated, which is the characteristics of cGNS. In addition, LNA and 2′-O-methylated MB described above could be combined with our system. In this study, we demonstrates the feasibility that cationized gelatin nanospheres incorporating MB functioned well to visualize the cell apoptosis. To our knowledge, this is the first time to visualize the cell apoptosis using MB. In the near future, the sustained intracellular release of MB will be achieved by controlling the degradability of gelatin nanospheres. This MB release may be a powerful tool to realize a prolonged visualization of apoptosis and other biological functions. The time profile of intracellular fluorescence should be evaluated in terms of intracellular cGNS and MB fate for the further applications.

## Methods

### Materials

Gelatin with an isoelectric point of 9.0 and the weight-averaged molecular weight of 99,000, prepared by an acidic process of pig skin, was kindly supplied from Nitta Gelatin Inc., Osaka, Japan. Molecular beacons (MB) for mouse messenger RNA (mRNA) of glyceraldehyde-3-phosphate dehydrogenase (GAPDH) and caspase-3 were designed by NIPPON GENE Co., Ltd, Tokyo, Japan, and synthesized by Eurogentec S.A., Seraing, Belgium. Target oligonucleotides of DNA for MB (GAPDH specific and caspase-3 specific sequence) were synthesized by Hokkaido System Science Co., Ltd, Sapporo, Japan. Glutaraldehyde (GA, 25 wt% in water), glycine, concentrated hydrochloric acid (HCl), acetone, and 1-ethyl-3-(3-dimethylaminopropyl) carbodiimide hydrochloride salt (EDC) were purchased from Nacalai Tesque. Inc., Kyoto, Japan. Spermine was purchased from Sigma-Aldrich Inc., St. Louis, MO, USA. The reagents were used without further purification.

### Preparation of cationized gelatin

According to the preparation procedure previously reported^23^, the carboxyl groups of gelatin were chemically converted by introducing amino groups to allow gelation to cationize. Spermine was added at a molar ratio of 50 to the carboxyl groups of gelatin into 50 ml of double-distilled water (DDW) containing 2.0 g of gelatin. Immediately after that, the solution pH was adjusted to 5.0 by adding 11 M HCl aqueous solution. EDC was added at a molar ratio of 3 to the carboxyl groups of gelatin. The reaction mixture was agitated at 40 °C for 18 hr, and then dialyzed against DDW for 3 days at room temperature. The dialyzed solution was freeze-dried to obtain a cationized gelatin. To determine the percentage of amino groups introduced into gelatin, the conventional 2,4,6-trinitrobenzene sulfonic acid (TNBS, Wako Pure Chemical Industries, Ltd., Osaka, Japan) method was performed^49^. The percentage was 44.8 mole% per the carboxyl groups of gelatin.

### Preparation of cationized gelatin nanospheres (cGNS) incorporating molecular beacons (MB)

Cationized gelatin nanosheres (cGNS) were prepared by the conventional coacervation method^26^. In brief, 1.25 ml of cationized gelatin aqueous solution (50 mg/ml) was warmed up to 40 °C. Next, 5 ml of acetone was added to the solution, and the coacervate was formed. GA (20 µl) were added, followed by chemically crosslinking cGNS for 6 hr. For the blocking of aldehyde groups unreacted, 2 ml of glycine aqueous solution (0.5 M) was added. The resulting solution was agitated overnight at 40 °C and the residual acetone was evaporated. cGNS were collected by the centrifugation of 14,000 rpm for 30 min at 25 °C and resuspended in DDW. The centrifugation and resuspension were repeated 3 times.

Two types of molecular beacons (MB) for GAPDH (GAP MB) and caspase-3 (casp3 MB) were used in this study. MB and cGNS were mixed at various ratios (2, 10, 20, 30, and 40 pmole/µg cGNS) and incubated for 15 min at room temperature. The mixture was centrifuged at 14,000 rpm for 30 min at 25 °C and resuspended in DDW to obtain cGNS incorporating GAP MB (cGNS_GAPMB_) and casp3 MB (cGNS_casp3MB_).

### Radiolabeling of MB

MB were radiolabeled with ^125^I^50^ with slight modification. Briefly, 5 µl of MB (10 µM) were incubated at 60 °C for 50 min with 2 µl of 0.3 mM Na_2_SO_3_, 5 µl of Na^125^I, and 5 µl of 4 mM TlCl_3_. Mixed 100 µl of 0.1 M Na_2_SO_3_, and 900 µl of 0.1 M NaCl, 50 mM Tris, and 1 mM ethylenediaminetetra acetic acid (EDTA) were added to the solution. After the incubation at 60 °C for 30 min, free ^125^I was removed by gel filtration on the PD-10 column (GE Healthcare Bio-Sciences Corp., Piscataway, NJ). The radioactivity of ^125^I was measured using a gamma counter (Auto Well Gamma System ARC-380 CL, Aloka Co., Ltd, Tokyo, Japan).

### Characterization of cGNS with or without MB incorporation

cGNS, cGNS_GAPMB_, and cGNS_casp3MB_ were resuspended in 10 mM phosphate buffered-saline solution (PBS, pH7.4), and the apparent size of nanospheres was measured by dynamic light scattering (DLS, Zetasizer Nano-ZS, Malvern Instruments Ltd., Worcestershire, UK). On the other hand, nanospheres were resuspended in 10 mM phosphate buffer solution (PB, pH7.4), and the zeta potential was measured by electrophoresis light scattering (ELS, Zetasizer Nano-ZS, Malvern Instruments Ltd., Worcestershire, UK). The amount of MB incorporated in cGNS was determined by the radioactivity of cGNS_GAPMB_ and cGNS_casp3MB_ prepared with the ^125^I-labeled MB. The experiment was independently performed 3 times for each samples unless otherwise mentioned.

### Hybridization Assay

Various concentrations of target oligonucleotides (GAPDH specific and caspase-3 specific, 0, 50, 100, 500, 1000, and 5000 nM) and free MB (GAP MB and casp3 MB, 100 nM) or cGNS incorporating MB (cGNS_GAPMB_ and cGNS_casp3MB_, 100 nM of MB) were mixed in Hybridization Buffer (20 mM Tris-HCl buffer containing 50 mM KCl and 5 mM MgCl_2_, pH8.0). After the incubation of 1 hr at room temperature under light protection, the fluorescent intensity was measured by Multi-mode Microplate Reader (SpectraMax i3x, Molecular Devices Japan Co., Ltd., Tokyo, Japan).

### Evaluation of nuclease stability

The stability of free MB and cGNS incorporating MB against DNase I (QIAGEN, Hilden, Germany) was evaluated. Various concentrations of DNase I (0, 0.5, 1, 5, 10, 15, 20, 30, and 40 U/ml) and free MB (GAP MB and casp3 MB, 100 nM) or cGNS incorporating MB (cGNS_GAPMB_ and cGNS_casp3MB_, 100 nM of MB) were mixed in PBS. After the incubation of 15 min at 37 °C in a condition of light protection, the fluorescent intensity was measured by Microplate Reader described above.

### Cell culture experiments

KUM6 of a mouse bone marrow-derived mesenchymal stem cell was purchased from JCRB Cell Bank (National Institute of Biomedical Innovation, Health and Nutrition, Osaka, Japan). The cells were cultured in Iscove’s Modified Dulbecco’s Medium (IMDM, GIBCO Lifetechnologies Co., Carlsbad, CA, USA) containing 10 vol% bovine fetal calf serum (FCS, Hyclone laboratories, Inc., Utah, UT, USA) and 1 vol% penicillin and streptomycin at 37 °C in a 5% CO_2_-95% air atmospheric condition. The cells were detached with 0.25 wt% trypsin-containing 1 mM EDTA solution (Nacalai Tesque. Inc., Kyoto, Japan), and continued to culture in 100 mm cell culture dish (Corning Inc., Corning, NY) to allow to grow until to 80% confluency.

### Evaluation of cell viability after incubation with cGNS incorporating MB

Cells were seeded into each well of 96 well multi-dish culture plate (Corning Inc., Corning, NY) at a density of 1 × 10^4^ cells/well and cultured for 24 hr. The medium was changed to OPTI MEM (GIBCO Lifetechnologies Co., Carlsbad, CA, USA), and then cGNS_GAPMB_ or cGNS_casp3MB_ (1, 5, 10, 15, and 20 µg/ml) were added to each well. The cell viability was evaluated using a cell counting kit (Nacalai Tesque. Inc., Kyoto, Japan). After the incubation of 3 hr with nanospheres, 10 µl of 2-(2-methoxy-4-nitrophenyl)-3-(4-nitrophenyl)-5-(2,4-disulfophenyl)-2H-tetrazolium (WST-8) solution was added to each well and further incubated for 1 hr. The absorbance of samples at 450 nm was measured by Microplate Reader. The percentage of cell viability was expressed as 100% for cells without nanospheres incubation.

### Evaluation of cellular internalization

Cells were seeded in each well of 6 well multi-dish culture plate (Corning Inc., Corning, NY) at a density of 5 × 10^4^ cells/well, and cultured for 24 hr. The medium was changed to the OPTI MEM, and then cGNS_GAPMB_ prepared with the ^125^I-labeled MB (1, 5, 10, 15, and 20 µg/ml) was added to each well. After the incubation of 3 hr with nanospheres, the medium was removed and cells were washed with PBS, and then the medium was added. The cells treated with nanospheres were collected by the trypsinization at 12 hr after nanospheres added. The amount of MB was determined by the radioactivity measurement.

For the inhibition of endocytosis, cells were similarly seeded in each well of 6 well multi-dish culture plate at a density of 5 × 10^4^ cells/well, and cultured for 24 hr. The medium was changed to the OPTI MEM, and cGNS_GAPMB_ (10 µg/ml, 153 pmole of MB) was added, followed by incubating for 3 hr at 4 °C. GAP MB (153 pmole) were similarly incubated with the cells to measure the amount of MB internalized into the cells based on the radioactivity.

### Evaluation of intracellular localization

To fluorescently label cGNS, fluorescein isothiocyanate isomer I (FITC, 200 µg/ml, Sigma-Aldrich Inc., St. Louis, MO, USA) and cGNS (5 mg/ml) were mixed in carbonate-bicarbonate buffer solution (0.1 M, pH9.6) at room temperature. After the incubation of 8 hr, the mixture was centrifuged at 14,000 rpm for 30 min at 25 °C and resuspended in DDW. The centrifugation and resuspension were repeated 3 times, and finally cGNS labeled with FITC (FITC-cGNS) were dispersed in DDW. FITC labeled-cGNS_GAPMB_ (FITC-cGNS_GAPMB_) were prepared by mixing GAP MB and FITC-cGNS at the same procedure described above.

Cells were seeded in a glass bottom dish of 35 mm in diameter (Matsunami Glass Industries Ltd., Tokyo, Japan) at a density of 5 × 10^4^ cells/dish, and cultured for 24 hr. After the medium change to the OPTI MEM, the cells were incubated with FITC-cGNS_GAPMB_ (10 µg/ml) for 3 hr, and further cultured for 9 hr. The lysosomes of cells treated with nanospheres were stained by LysoTracker Red (80 nM, Thermo Fisher Scientific Inc., Massachusetts, USA), followed by washed with PBS and fixed with 4 vol% paraformaldehyde for 20 min. The nuclei of cells were stained by 4′,6-diamidino-2-phenylindole (DAPI, 300 nM, Thermo Fisher Scientific Inc., Massachusetts, USA). The fluorescent images of cells were taken by a fluorescent microscopy BZ-X700 (KEYENCE Co., Ltd., Osaka, Japan).

### Fluorescent imaging of apoptosis

After the incubation of 12 hr with cGNS_GAPMB_ or cGNS_casp3MB_ (10 µg/ml), apoptosis was induced by camptothecin (Enzo Life Sciences, Inc., Farmingdale, NY)^51^. In brief, various concentrations of camptothecin (final concentrations of 1, 2, 5, 10, and 20 µM) were added to the cells, and cultured for 12 hr to induce the cell apoptosis. After the apoptosis induction, the cells were observed by the fluorescent microscopy. Six fluorescent images were taken at random and quantified by BZ-X Analyzer equipped with the microscope. The fluorescence of images was calculated according to the following equation:

$${\rm{Fluorescent}}\,{\rm{intensity}}={\rm{Fluorescent}}\,{\rm{Area}}\times {\rm{Mean}}\,{\rm{Fluorescent}}\,{\rm{Intensity}}$$

### Apoptosis analysis

Apoptosis of cells was evaluated by flow cytometry analysis using FITC Annexin V Apoptosis Detection Kit I (Becton Dickinson, Franklin Lakes, NJ) according to the manufacture’s protocol. Briefly, cells were seeded in each well of 6 well multi-dish culture plate at a density of 1 × 10^5^ cells/well, and cultured for 24 hr. Camptothecin (final concentrations of 0, 1, 2, 5, 10, and 20 µM) were added to the cells, and cultured for 12 hr. The cells were collected by the trypsinization and washed with cold PBS twice. Finally, the cells were suspended in 10 mM HEPES/NaOH solution containing 140 mM NaCl and 2.5 mM CaCl_2_ (pH 7.4), and FITC-conjugated annexin V and propidium iodide (PI) were added to the suspension. The cell suspension was analyzed on fluorescence activated cell sorting FACSCanto II (Becton Dickinson, Franklin Lakes, NJ).

### mRNA expression analysis

Cells were seeded in each well of 6 well multi-dish culture plate at a density of 1 × 10^5^ cells/well, and induced apoptosis by camptothecin at the same procedure described above. After the apoptosis induction for 12 hr, the total RNA was extracted using RNeasy Plus Mini Kit (QIAGEN, Hilden, Germany) according to the manufacture’s instructions. Complementary DNA (cDNA) was synthesized using a SuperScript VILO cDNA synthesis kit (Thermo Fisher Scientific Inc., Massachusetts, USA). The cDNA (100 ng, 1 µl), forward and reverse primers (10 µM, each 0.5 µl), and 12.5 µl of Power SYBR Green PCR Master Mix (Applied Biosystems, Foster City, CA) were mixed, and real-time polymerase chain reaction (PCR) was performed on a Prism 7500 real-time PCR thermal cycler (Applied Biosystems, Foster City, CA). The sequences of primers used were listed in Table 1. The following PCR conditions were used: 95 °C for 10 min, followed by 40 cycles of 95 °C for 15 s and 60 °C for 1 min. GAPDH was used as a housekeeping gene, and the expression level was analyzed by ΔΔ*C*_*t*_ method comparing with the untreated cells.

**Table 1:** MB, target oligonucleotides, and PCR primers used.

MB, target nucleotides, and primers	sequences (5′ to 3′)
GAPDH MB	[Cy5]-CTGGTAATCCGTTCACACCGACCTTCACCAG-[BHQ-2]
caspase-3 MB	[FAM]-GTCACATACAGGAAGTCAGCCTCCACCGTGAC-[BHQ-1]
GAPDH specific target	TGGTGAAGGTCGGTGTGAACGGATT
caspase-3 specific target	CGGTGGAGGCTGACTTCCTGTATG
GAPDH forward	AACTTTGGCATTGTGGAAGG
GAPDH reverse	GGAGACAACCTGGTCCTCAG
caspase-3 forward	TGTCATCTCGCTCTGGTACG
caspase-3 reverse	AAATGACCCCTTCATCACCA

GAPDH: glyceraldehyde-3-phosphate dehydrogenase, BHQ: black hole quencher, underline: stem structure.

### Statistical analysis

All the statistical data were expressed as the mean ± standard deviations. The data were analyzed by Tukey-Kramer paired comparison test and the statistical significance was accepted at p < 0.05.

## Electronic supplementary material

Supplementary Information
