# Supplementary material for: Preparation of cationized gelatin nanospheres incorporating molecular beacon to visualize cell apoptosis
Source: Sci Rep. 2018 Oct 4;8:14839. doi: 10.1038/s41598-018-33231-2 (PMC6172245; doi:10.1038/s41598-018-33231-2)
Supplement: Supplementary file 1 — Supplementary Information [file 41598_2018_33231_MOESM1_ESM.pdf]

Supplementary Information

Preparation of cationized gelatin nanospheres incorporating molecular beacon to visualize cell apoptosis

Yuki Murata, Jun-ichiro Jo, and Yasuhiko Tabata*

Laboratory of Biomaterials, Institute for Frontier Life and Medical Sciences, Kyoto University, 53 Kawara-cho Shogoin, Sakyo-ku Kyoto 606-8507, Japan

*Corresponding author. Laboratory of Biomaterials, Department of Regeneration Science and Engineering, Institute for Frontier Life and Medical Sciences, Kyoto University, 53 Kawara-cho Shogoin, Sakyo-ku, Kyoto 606-8507, Japan.

Tel.: +81 75 751 4121
Fax: +81 75 751 4646
E-mail: yasuhiko@infront.kyoto-u.ac.jp

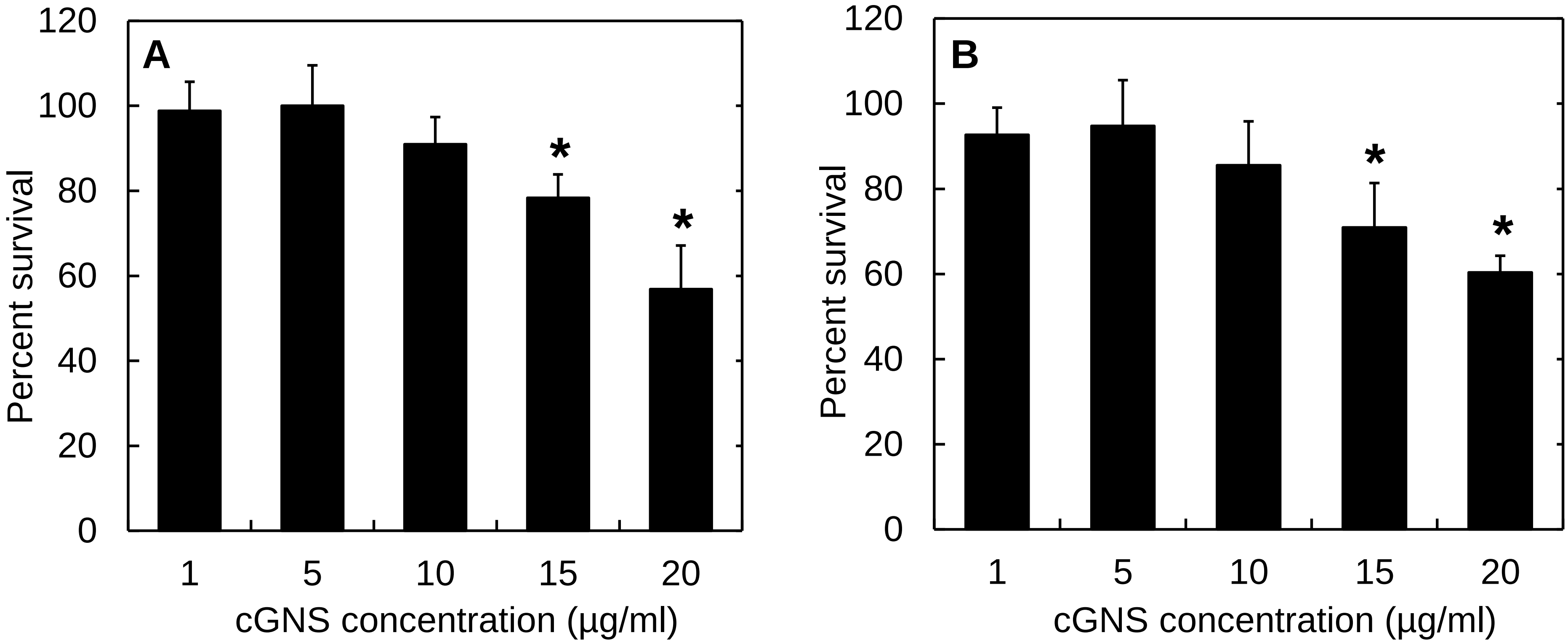

Supplementary Figure 1

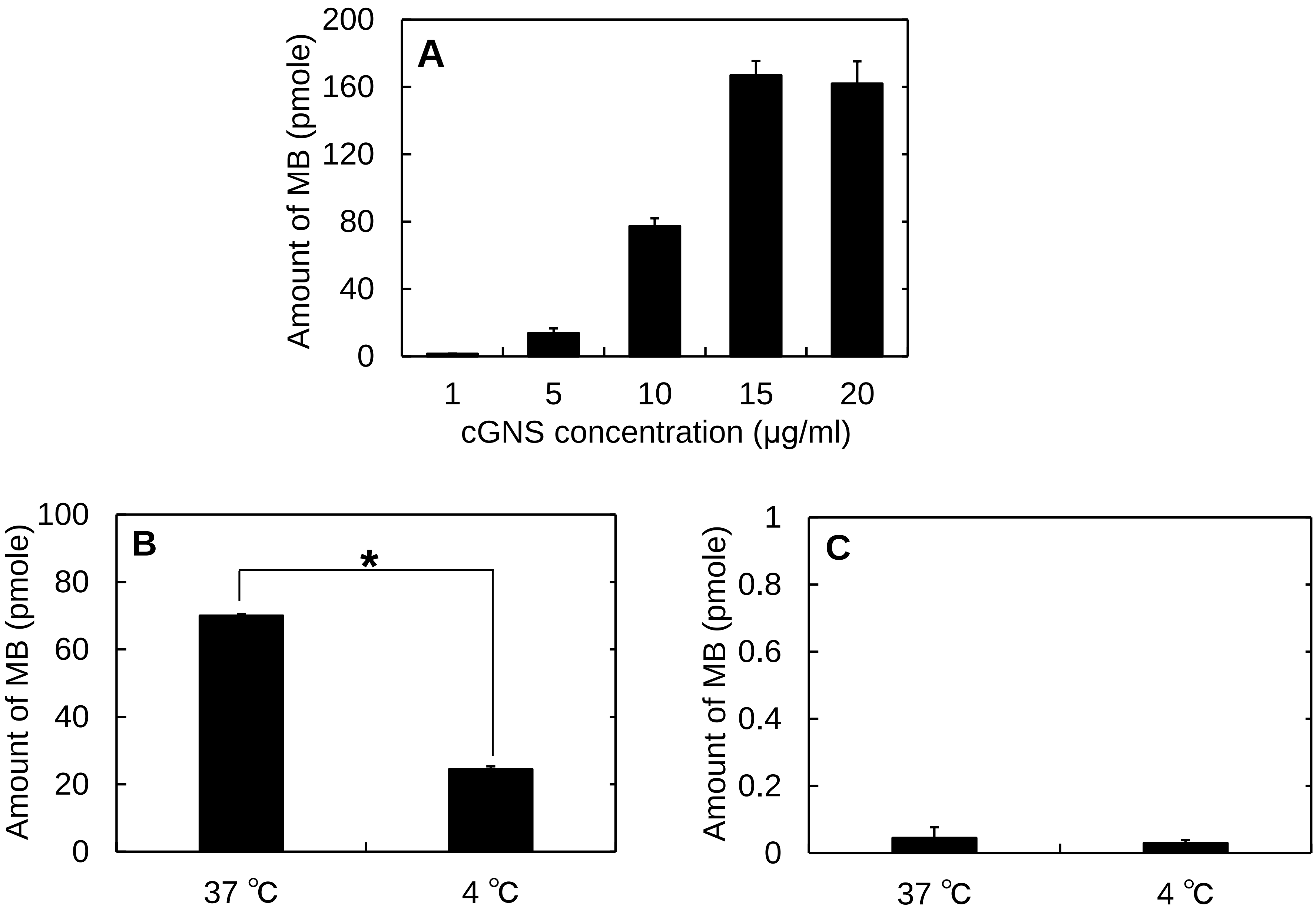

Supplementary Figure 2

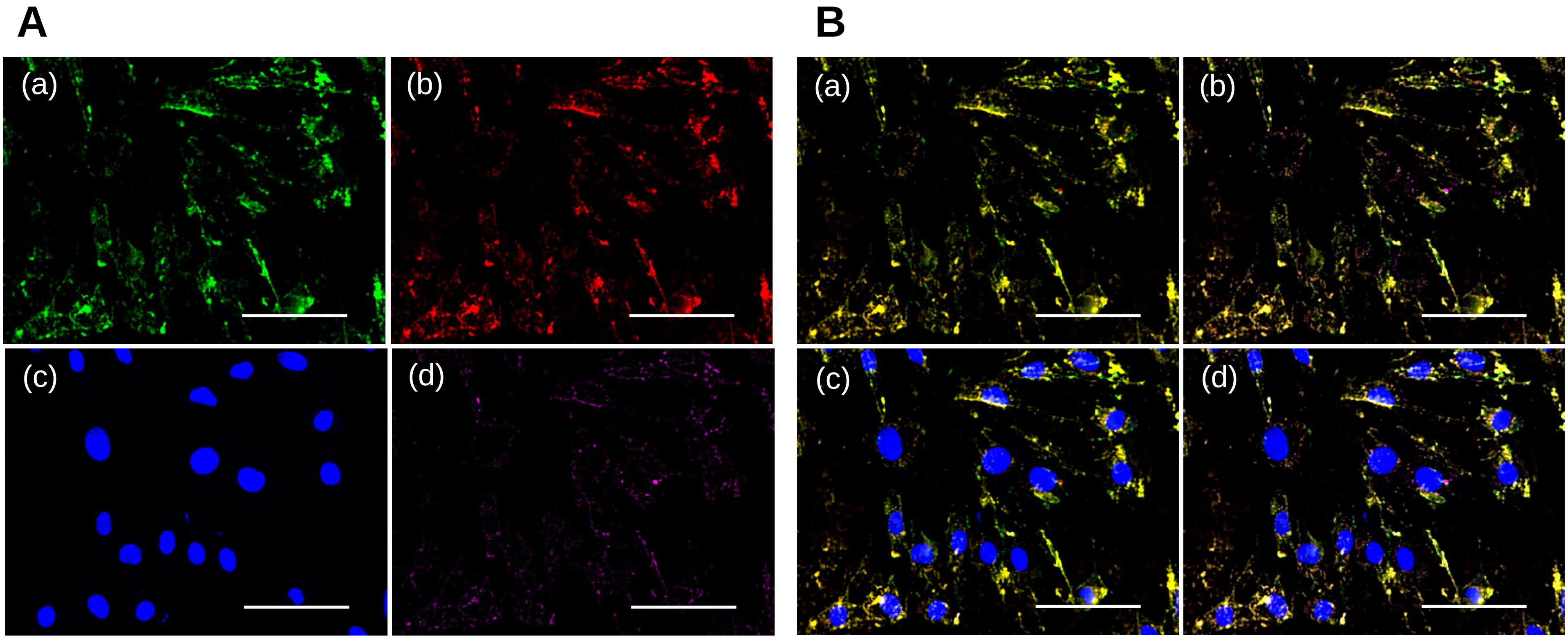

Supplementary Figure 3

Supplementary Figure Legends

Supplementary Figure 1. Viability of cells incubated with different concentrations of $cGNS_{GAP\ MB}$ (A) and $cGNS_{casp3\ MB}$ (B). The viability of cells without incubation with cGNS was expressed 100%. *, $p < 0.05$; significant against the percent survival of cells without cGNS.

Supplementary Figure 2. Cellular internalization of free GAP MB and $cGNS_{GAP\ MB}$. (A) The effect of cGNS concentrations on the amount of MB internalized into the cells. (B) The amount of MB internalized into cells after incubation with $cGNS_{GAP\ MB}$ (10 μg/ml, 153 pmole of MB) at 37 and 4 ℃. (C) The amount of MB internalized into cells after incubation with free GAP MB (153 pmole) at 37 and 4 ℃. *, $p < 0.05$; significant against the amount at 37 ℃.

Supplementary Figure 3. Fluorescent microscopic images of FITC-$cGNS_{GAP\ MB}$ internalized into cells. (A) Fluorescent images of cells after incubation with FITC-$cGNS_{GAP\ MB}$ (10 μg/ml): (a) Green: cGNS, (b) Red: GAP MB, (c) Blue: nuclei, and (d) Magenta: lysosomes. (B) Merged fluorescent images: (a) cGNS and GAP MB, (b) cGNS, GAP MB, and lysosomes, (c) cGNS, GAP MB, and nuclei, and (d) cGNS, GAP MB, nuclei, and lysosomes. Scale bar is 100 μm.
